# Differentiating patients admitted primarily due to coronavirus disease 2019 (COVID-19) from those admitted with incidentally detected severe acute respiratory syndrome corona-virus type 2 (SARS-CoV-2) at hospital admission: A cohort analysis of German hospital records

**DOI:** 10.1017/ice.2024.3

**Published:** 2024-06

**Authors:** Ralf Strobl, Martin Misailovski, Sabine Blaschke, Milena Berens, Andreas Beste, Manuel Krone, Michael Eisenmann, Sina Ebert, Anna Hoehn, Juliane Mees, Martin Kaase, Dhia J. Chackalackal, Daniela Koller, Julia Chrampanis, Jana-Michelle Kosub, Nikita Srivastava, Fady Albashiti, Uwe Groß, Andreas Fischer, Eva Grill, Simone Scheithauer

**Affiliations:** 1 Institute for Medical Information Processing, Biometrics and Epidemiology, Faculty of Medicine, LMU Munich, Muenchen, Germany; 2 German Center for Vertigo and Balance Disorders, LMU University Hospital, LMU Munich, Muenchen, Germany; 3 Department of Infection Control and Infectious Diseases, University Medical Center Goettingen, Goettingen, Germany; 4 Emergency Department, University Medical Center Goettingen, Goettingen, Germany; 5 Institute for Hygiene and Microbiology, University of Wurzburg, Wurzburg, Germany; 6 Infection Control and Antimicrobial Stewardship Unit, University Hospital Wurzburg, Wurzburg, Germany; 7 Medical Data Integration Center, LMU University Hospital, LMU Munich, Muenchen, Germany; 8 Institute of Medical Microbiology and Virology, University Medical Center Goettingen, Goettingen, Germany; 9 Institute for Clinical Chemistry, University Medical Center Goettingen, Goettingen, Germany

## Abstract

**Objective:**

The number of hospitalized patients with severe acute respiratory syndrome coronavirus type 2 (SARS-CoV-2) does not differentiate between patients admitted due to coronavirus disease 2019 (COVID-19) (ie, primary cases) and incidental SARS-CoV-2 infection (ie, incidental cases). We developed an adaptable method to distinguish primary cases from incidental cases upon hospital admission.

**Design:**

Retrospective cohort study.

**Setting:**

Data were obtained from 3 German tertiary-care hospitals.

**Patients:**

The study included patients of all ages who tested positive for SARS-CoV-2 by a standard quantitative reverse-transcription polymerase chain reaction (RT-PCR) assay upon admission between January and June 2022.

**Methods:**

We present 2 distinct models: (1) a point-of-care model that can be used shortly after admission based on a limited range of parameters and (2) a more extended point-of-care model based on parameters that are available within the first 24–48 hours after admission. We used regression and tree-based classification models with internal and external validation.

**Results:**

In total, 1,150 patients were included (mean age, 49.5±28.5 years; 46% female; 40% primary cases). Both point-of-care models showed good discrimination with area under the curve (AUC) values of 0.80 and 0.87, respectively. As main predictors, we used admission diagnosis codes (ICD-10-GM), ward of admission, and for the extended model, we included viral load, need for oxygen, leucocyte count, and C-reactive protein.

**Conclusions:**

We propose 2 predictive algorithms based on routine clinical data that differentiate primary COVID-19 from incidental SARS-CoV-2 infection. These algorithms can provide a precise surveillance tool that can contribute to pandemic preparedness. They can easily be modified to be used in future pandemic, epidemic, and endemic situations all over the world.

Throughout the coronavirus disease 2019 (COVID-19) pandemic there has been an urgent need for indicators of pandemic burden of disease to guide public health decisions. Initially, in most countries worldwide, the national or regional incidence rate based on the number of patients who tested positive for severe acute respiratory syndrome coronavirus type 2 (SARS-CoV-2) served as the main indicator for the pressure on healthcare systems.^1^

The hospitalization rate is calculated as the rate of hospitalized patients with a positive test among all hospitalized patients and was supposed to better reflect this pressure. Starting in 2021, the hospitalization rate was established as a suitable indicator, for example, in Germany, facilitated by a uniform admission screening by reverse-transcription polymerase chain reaction (RT-PCR) for all patients admitted to an acute-care hospital.^1,2^ Universal admission testing of patients contributed to an increase of the hospitalization rate, diminishing the validity of the hospitalization rate as a measure of disease burden. Initially, most hospitalized patients with confirmed SARS-CoV-2 infection presented with severe symptoms of the coronavirus disease 2019 (COVID-19).^3^ This was reasonable because patients were only admitted to elective hospital procedures if they presented a negative RT-PCR test result. With increasing population immunity, either from previous infections or through vaccination, testing positive for SARS-CoV-2 has become less indicative of a severe disease course, leading to a potential overestimation of the actual burden.^4–6^ For example, in a study from the Netherlands, only 45% of all adult patients who tested positive for SARS-CoV-2 were primarily admitted due to COVID-19.^7^ Other studies have raised the need to include disease severity for better surveillance. They defined severe disease as having received SARS-CoV-2 therapeutics like oxygen supplementation or specific medication, namely corticosteroids, or a clear sign of a related deficit, such as a decreased oxygen saturation during hospital stay.^3,8,9^ Nonetheless, the prevailing definitions used to determine severity level of COVID-19 hospitalizations have led to invalid case counts and outcomes.^10^

All potential measures demand standardized and timely access to the electronic health record (EHR) for effective surveillance during a dynamic pandemic situation. A prerequisite for this surveillance is the existence of well-established and nationwide standardized EHR, which is often lacking. For instance, in German hospitals, medication data are inconsistently documented in different hospitals, sometimes even among clinical departments of a single hospital. One potential solution is to incorporate mortality data. However, until now, no standardized criteria have been available to distinguish deaths caused by COVID-19 from deaths that coincide with COVID-19. Comparing international and national statistics is difficult because the determination of the cause of death varies among countries, over time, and among individual practitioners and local institutions.^11,12^

We hypothesized that the hospitalization rate still has the potential to guide policy makers to escalate or de-escalate infection prevention and control and public health measures if it is solely based on the number of patients mainly treated for COVID-19–related symptoms or to prevent a severe course in primary cases. Thus, the hospitalization rate should not count patients whose admission is unrelated to the presence of a SARS-CoV-2 infection (ie, incidental cases). Our new approach provides a more nuanced strategy for both public health interventions and hospital planning. For instance, primary cases may require distinct treatment protocols compared to incidental cases, which could affect resource allocation at the hospital level.

In this study, we developed a model to differentiate between primary and incidental cases based on data retrieved from the EHR that could enable automated reporting of primary cases irrespective of clinical or hospital infection prevention and infection control measurements to inform decision makers in a timely manner. Specifically, we developed 2 distinct models: (1) a point-of-care model that is based solely on data easily available, ideally in the first 24 hours and (2) a more extended point-of-care model based on parameters that are available within the first 24–48 hours after admission.

## Methods

### Overview

We applied an exploratory, sequential, mixed-methods design integrating qualitative evidence from experts and the systematic review of the literature with the analysis of real-world data from the hospital EHR.^13^ We conducted 3 preparatory studies: (1) an online survey among German healthcare professionals, (2) semistandardized qualitative interviews with international experts, and (3) a systematic rapid literature review. The details of these 3 studies are being published elsewhere (M. Misailovski, et al, unpublished data, 2023).

### Study design and participants

To establish the model, we used data from 2 German tertiary-care hospitals: University Medical Center Goettingen (UMG) and University Hospital Wurzburg (UKW). The applicability of the model was tested using data from the University Hospital Munich (UHM). Patients of all ages tested positive for SARS-CoV-2 by a standard quantitative RT-PCR assay prior to or within the first 3 days after hospital admission were included. All admissions between January 1 and June 30, 2022, were considered with an exception for patients admitted to psychiatric or psychosomatic facilities (UMG). Data from UHM included all patients until December 12, 2022. Data protection clearance and approval was obtained from the local ethics committees at UMG (no. 10/8/22), UKW (no. 20221205 02), and UHM (no. 22-0912).

## Variables

### Primary case versus incidental case

At UMG and UKW, an initial meeting was organized to discuss data collection. A 3-member team comprising a study nurse, a medical doctor, and an experienced senior consultant classified patients as primary or incidental cases based on the medical record and clinical reasoning. For disagreements, consensus was achieved after discussion with the senior consultant. All team members were instructed to follow the definitions of primary and incidental cases. At the UHM, expert classification was not available.

A primary case was defined as admission primarily for treating COVID-19 symptoms or anticipating severe COVID-19 in immunocompromised patients (eg, hemato-oncological). Cases with admissions unrelated to SARS-CoV-2 infection (eg, traumatic injury) were classified as incidental.

### Predictors

Predictors in the models included both clinical indicators (eg, symptoms and test results) and risk factors (eg, comorbidities and underlying health conditions). The patient records within the clinical information systems of the UMG were utilized to extract data for a predefined set of variables. Data were manually compiled due to the inconsistent way data were stored. For the UKW data set, pseudonymized data were extracted from the clinical information system combined with data from the COVID-19 surveillance database of the hospital. Clinical parameters and therapeutic treatment could not be included for the UKW data.

Data from UHM were retrospectively analyzed based on the clinical routine data integrated in the Medical Data Integration Center (MeDIC).

We included 51 predictors that emerged as potentially relevant from the 3 preparatory studies. We categorized admission wards as nonsurgical, surgical, or intensive care unit. Oxygen therapy was defined as any oxygen therapy via nasal canula or face mask. C-reactive protein (CRP) was measured in milligrams per liter (mg/L). According to the threshold for each test system, a standardized recalculation in genome equivalent copies per milliliter was performed. Viral load is reported here as the logarithm to base 10 of the quantity of the SARS-CoV-2 viral particles in the test sample. Admission diagnoses were assigned within 24 hours and were based on the *International Statistical Classification of Diseases, 10th Revision, German Modification* (ICD-10-GM). All ICD-10 diagnoses indicating primary cases considered relevant in the preparatory studies were included. Next, we conducted a bivariate analysis of the ICD-10 diagnoses and the outcomes.

Each ICD-10 diagnosis with >60% of primary cases and a frequency of >4 was considered relevant, and the list of relevant diagnoses was extended accordingly. The selection of the 60% threshold was informed by the research of Klann et al^14^ and was chosen for its practical suitability. Please refer to the Supplementary Materials (online) for details regarding results of the preparatory studies, all variables, and their transformations.

### Statistical analysis

We developed a point-of-care model and an extended point-of-care model. The point-of-care model contained only data available shortly after admission that were easy to retrieve from the EHR. These data included the admission diagnoses, the ward of admission, age, and sex. The extended point-of-care model included additional laboratory tests (eg, viral load), and clinical variables. The point-of-care model was developed using UKW data and was validated using UMG data. The extended point-of-care model was developed using UMG data.

### Statistical modeling

We present mean and standard deviation for continuous variables and absolute and relative percentages for categorical variables. The point-of-care model was developed using logistic regression. We report the exponential of the coefficients as the odds ratio (OR). The extended point-of-care model was developed using classification and regression trees (CART). We chose CART because it imitates human decision making by breaking down the decisions into distinct binary choices, while also effectively managing missing data through the utilization of surrogate variables.^15–17^ Surrogate variables are selected due to high correlation with the splitting variable and are employed to establish the direction of the split when encountering missing values. Random forests were used to measure variable importance of all clinical variables.^18^ We calculated sensitivity, specificity, and overall accuracy for each model.^19^ Calibration was assessed visually and using the Brier score with higher Brier scores representing worse calibration.^20^ Receiver operating characteristics (ROC) curves and the respective area under the curve (AUC) were calculated to describe discrimination. The AUC has a range of 0 to 1, with AUC values >0.5 indicating that the model discriminates better than chance. Because the models were constructed using distinct data sets, model comparison was based on measurements that were not dependent on sample size. Thus, model comparison was based on sensitivity, specificity, and overall accuracy. We have reported the 95% confidence intervals for each estimate. Statistical significance was set at a 2-tailed 5% level. R version 4.2.1 software was used for descriptive analyses and model development.^21^

## Results

**Figure 1.**
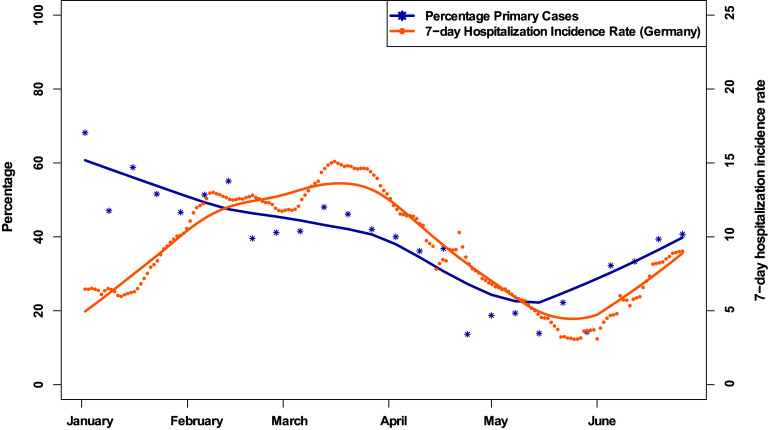
Visualization of the updated hospitalization rate in 2 German university hospitals during January–June 2022 (n = 1,150). The blue line indicates the percentage of primary cases among all hospitalized patients with confirmed SARS-CoV-2 infection in 2022. The yellow line indicates the hospitalization rate of patients with confirmed SARS-CoV-2 infection per 100,000 inhabitants per 7 days in Germany. Both curves have been smoothed using locally weighted regression.

In total, 1,150 patients from UKW and UMG were included: mean age, 49.5 (SD, 28.5); 46% female; 40% primary cases (Table 1 and Supplementary Table S4 online). Figure 1 shows the 7-day hospitalization rate in Germany in comparison to the percentage of primary cases among all hospitalized patients at UMG and UKW for the study period.

**Table 1.** Baseline Characteristics of the Study Population from University Hospital Wurzburg (UKW) and University Medical Center Goettingen (UMG)^a^

Levels	All (N=1,150), No. (%)^b^	Primary Case (n=462), No. (%)^b^	Incidental Case (n=688), No. (%)^b^
Age, median y (SD)	49.5 (±28.5)	46.0 (±31.7)	51.8 (±25.9)
**Age group**			
Infants (<1 y)	70 (6)	51 (11)	19 (3)
Preschool (1–5 y)	83 (7)	53 (11)	30 (4)
School (6–12 y)	42 (4)	20 (4)	22 (3)
Adolescent (13–17 y)	32 (3)	14 (3)	18 (3)
Adults (≥18 y)	923 (80)	324 (70)	599 (87)
**Ward of admission**			
Intensive care unit	164 (14)	114 (25)	50 (7)
Nonsurgical ward	639 (56)	232 (50)	407 (59)
Surgical ward	347 (30)	116 (25)	231 (34)
Need for intensive care during hospital stay	147 (13)	89 (19)	58 (8)
In-hospital mortality	54 (5)	36 (8)	18 (3)
LOS, median d (±SD)	7.7 (±12)	7.6 (±10.1)	7.8 (±13.1)
**Vaccination status**			
No vaccination	323 (28)	187 (40)	136 (20)
Vaccinated against SARS-CoV-2	607 (53)	231 (50)	376 (55)
Unknown	220 (19)	44 (10)	176 (26)
**Timing of RT-PCR test**			
Before admission	12 (1)	4 (1)	8 (1)
At admission	1026 (89)	408 (88)	618 (90)
<1–3 d after admission	112 (10)	50 (11)	62 (9)
**Variables used in the extended model (UMG data with n = 344)**			
Oxygen therapy	56 (16)	51 (26)	5 (3)
Logarithm to base 10 of viral load (±SD)^c^	5.9 (±2.2)	6.5 (±1.9)	5.0 (±2.4)
C-reactive protein (mg/L) (±SD)^d^	44.2 (±75.6)	47.5 (±77.8)	39.2 (±72.3)

Note. RT-PCR, reverse transcriptase-polymerase chain reaction; SARS-CoV-2, severe acute respiratory syndrome coronavirus type 2.

a Primary cases are those admitted to the hospital because of acute COVID-19. Incidental cases are those patients whose admission to the hospital was unrelated to their SARS-CoV-2 infection.

b Units unless otherwise specified.

c Data were missing for 10 patients.

d Data were missing for 34 patients.

### ICD-10 diagnoses

Based on the results of the preparatory studies, the following ICD-10 codes were considered indicative for a primary case: essential (primary) hypertension (I10); acute upper respiratory infections of multiple and unspecified sites (J06); viral pneumonia, not elsewhere classified (J12); pneumonia, organism unspecified (J18); adult respiratory distress syndrome (J80); respiratory failure, not elsewhere classified (J96); cough (R05); other symptoms and signs involving the circulatory and respiratory systems (R09); disturbances of smell and taste (R43); fever of other and unknown origin (R50); diseases of uncertain etiology, assigned and unassigned codes (U07); and multisystem inflammatory syndrome associated with COVID-19 (U10).

The following admission diagnoses were added according to their occurrence in the data set: viral and other specified intestinal infections (A08), viral infection of unspecified site (B34), other infectious diseases (B99), pulmonary embolism (I26), other respiratory disorders (J98), dyspnea (R06), malaise and fatigue (R53), and carrier of infectious disease (Z22) (see Supplementary Table S3 online).

### Point-of-care model

The model based on the variables age, sex, admission diagnosis, and ward of admission yielded high discriminative ability with an AUC of 0.86 (95% confidence interval [CI], 0.83–0.89) and good calibration performance, with a Brier score of 0.12 (95% CI, 0.11–0.14). Having at least 1 relevant ICD-10-GM diagnosis (OR, 23.98; *P* < .01), admission to a surgical ward (OR, 0.25; *P* = .01), and age ≥18 years (OR, 0.12; *P* < .01) were significantly associated with the outcome (Table 2). Applying this model to the validation data set of the UMG yielded an AUC of 0.80 (95% CI, 0.76–0.85) and a Brier score of 0.19 (95% CI, 0.16–0.22).

**Table 2.** Results for the Point-of-Care Model^a^

Variable	OR (95% CI)	*P* Value
Intercept	2.39 (0.72–7.99)	.15
**Age (reference, infants)**		
Preschool (1–5 y)	0.55 (0.19–1.55)	.26
School (6–12 y)	0.37 (0.12–1.16)	.09
Adolescent (13–17 y)	0.51 (0.15–1.73)	.29
Adults (≥18 y)	0.12 (0.05–0.28)	<.01
Sex (reference = male)	1.11 (0.75–1.66)	.60
At least 1 relevant ICD-10-GM diagnosis (reference, none)	23.98 (15.78–37.22)	<.01
**Ward of admission (reference, ICU)**		
Nonsurgical ward	0.50 (0.22–1.11)	.09
Surgical ward	0.25 (0.10–0.66)	.01

Note. OR, odds ratio; CI, confidence interval; ICD-10-GM, *International Statistical Classification of Diseases and Related Health Problems*.

a We report ORs and their respective 95% CIs. ORs >1 indicate variables indicative of a primary case; ORs <1 are indicative of an incidental case.

### Extended point-of-care model

**Figure 2.**
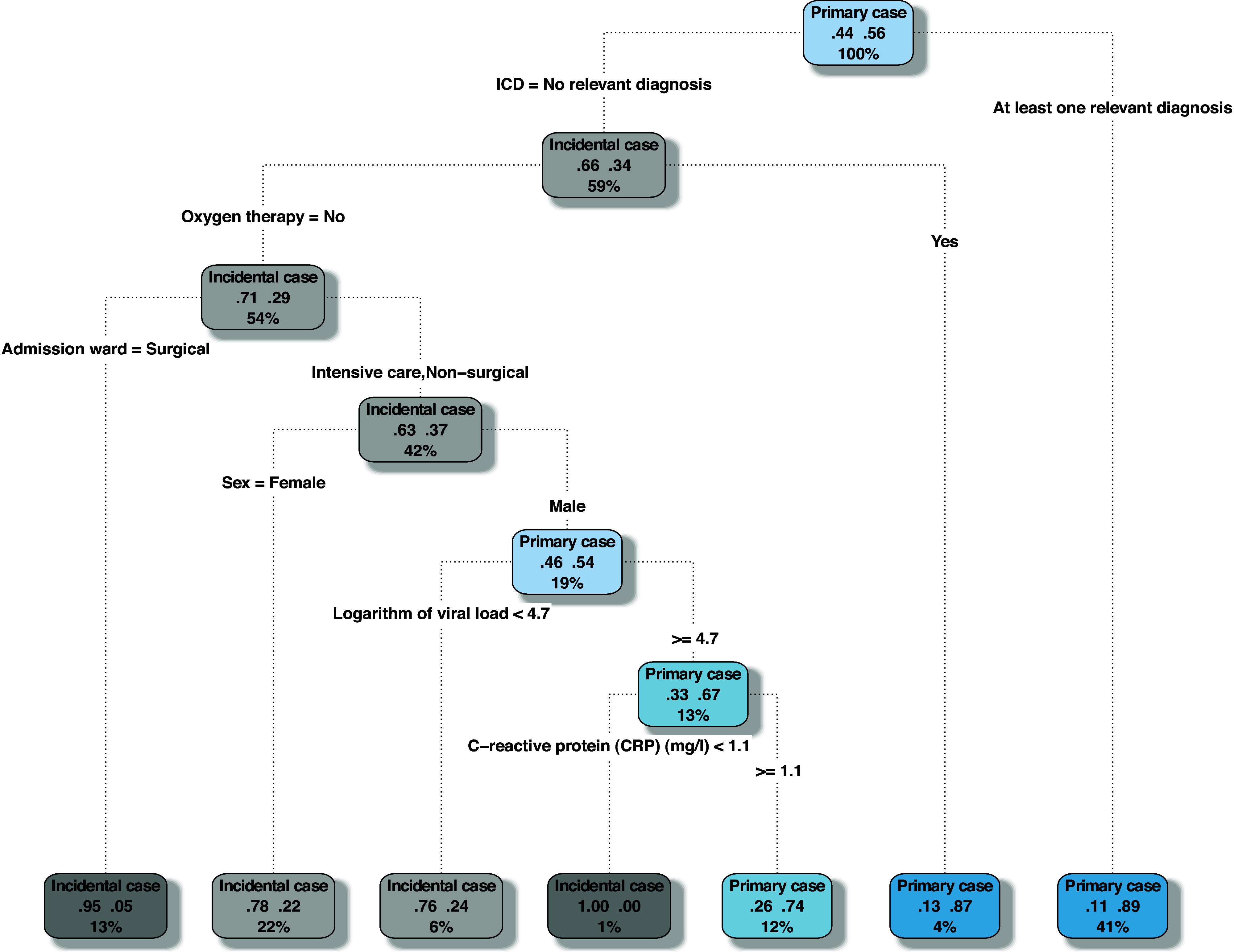
Decision tree based on UMG data: the extended point-of-care model. The tree was pruned by 10-fold cross validation. Blue nodes indicate subgroups with >50% primary cases. Grey nodes indicate >50% incidental cases, with title indicating the majority class, percentages of incidental and primary cases, and percentages of all patients in the respective nodes.

The extended point-of-care model was based on UMG data. Optimal split was obtained using admission diagnoses, sex, ward of admission, oxygen therapy, logarithm of viral load, and CRP levels. The resulting model yielded an AUC of 0.87 (95% CI, 0.83–0.91), sensitivity of 88% (95% CI, 82%–92%), specificity of 81% (95% CI, 73%–87%), accuracy of 85% (95% CI, 82%–88%), and Brier score of 0.12 (95% CI, 0.10–0.15) (Fig. 2). The respective ROC curves and calibrations plot are shown in the Supplementary Materials (online). We present a simplified version of the final decision algorithms in Table 3.

**Table 3.** Simplified Decision Algorithms

Included Variables	Sensitivity, %	Specificity, %	Accuracy, %
**Point-of-care models**			
1 or more ICD-10-GM code at admission	68	92	82
1 or more ICD-10-GM code at admission or intensive care	74	86	81
**Extended point-of-care model**			
Relevant ICD-10-GM code at admission or oxygen therapy or (nonsurgical ward and male and logarithm of viral load > 4.7; CRP ≥1.1 mg/L)	88	81	85

Note. ICD-10-GM, *International Statistical Classification of Diseases and Related Health Problems*; CRP, C-reactive protein.

### Variable importance

Running random forests, the 5 most important variables were admission diagnosis, viral load, leukocytes, CRP, and thrombocytes (see Supplementary Table S4 (online) for the 20 most important variables).

### Practical application

For assessing practical applicability, we applied the algorithm to 1,423 patients from UHM (mean age, 59.0 years; SD, 21.6; 43% female). Data were adapted in consultation with health experts at UHM. Classification as a primary or incidental case was based on the point-of-care model (Table 4).

**Table 4.** Characteristics of Patients of the University Hospital Munich (UHM)^a^

Levels	All (N=1,423), No. (%)^b^	Primary Case (n=668), No. (%)^b^	Incidental Case (n=750), No. (%)^b^
Sex, female	610 (43)	287 (43)	322 (43)
Age median y (±SD)	59.0 (±21.6)	61.4 (±22.6)	57.1 (±20.2)
At least 1 relevant ICD- 10-GM diagnosis	561 (39)	560 (84)	0 (0)
**Age in classes (y)**			
Infants (<1 y)	18 (1)	18 (3)	0 (0)
Preschool (1–5 y)	18 (1)	9 (1)	9 (1)
School (6–12 y)	17 (1)	8 (1)	9 (1)
Adolescent (13–17 y)	23 (2)	10 (1)	13 (2)
Adults (≥18 y)	1343 (95)	623 (93)	719 (96)
**Ward of admission**			
Intensive care unit	64 (4)	33 (5)	30 (4)
Nonsurgical	1,252 (88)	623 (93)	625 (83)
Surgical	107 (8)	12 (2)	95 (13)
Need for intensive care during hospital stay	241 (17)	94 (39)	145 (61)
Length of stay, median d (±SD)	9.5 (±11.6)	8.6 (±11)	10.2 (±12.1)

Note. ICD-10-GM, *International Statistical Classification of Diseases and Related Health Problems*.

a Classified into primary or incidental case based on the point-of-care model (5 patients could not be classified because of missing values in one of the predictor variables).

b Units unless otherwise specified.

## Discussion

We propose an algorithm differentiating incidental infections upon hospital admission from patients admitted due to COVID-19 disease. This measure may enable better pandemic preparedness to assist stakeholders in governance and resource allocation decisions and to guide public health measures. Admission diagnosis, the need for oxygen therapy, and distinct laboratory parameters, including leukocyte count, CRP levels, and mean corpuscular hemoglobin value were identified as valid and highly predictive variables that can be used for differentiation.

In contrast to recent studies proposing to base surveillance on disease severity identified by parameters gathered during the entire inpatient stay,^3,9^ we concentrated our analyses on data easily available at or shortly after admission. In this model, we used a combination of ICD-10 codes, ward of admission, and age to reach an acceptable discriminatory validity. This model could be improved by including variables for viral load, laboratory results, and the need for oxygen therapy. The predictive value of ICD-10 codes and laboratory tests confirms the findings of previous studies, such as a large study in the United States that used automated phenotyping of the EHR including retrospective diagnosis and presence or absence of laboratory tests.^14^ In contrast to this study, we included initially assigned ICD-10 codes, that is, diagnoses reflecting the initial clinical assessment at admission and laboratory test results. In line with literature, we found that ICD-10 codes that indicate clinical signs and symptoms (eg, unspecified respiratory symptoms and fever) were good initial predictors of primary COVID-19.^22^ In our study, high viral load was an important measure of infectiousness as well as an important indicator for primary COVID-19, which confirms previous findings regarding predictors of severe disease progression.^23^

As expected, in our study, low leucocyte count and elevated CRP levels were highly indicative of a primary case, as was higher mean corpuscular hemoglobin value.^24,25^ The need for oxygen therapy was a good indicator for primary cases, in line with current knowledge and treatment recommendations.^7,26^ Hypoxic respiratory disease has been mentioned as an indicator for disease severity in the context of vaccine effectiveness and as a differentiation criterion for incidental SARS-CoV-2 infection without clinically relevant COVID-19.^27,28^

The strengths of our study include the initial variable selection by formally triangulating studies involving experts and a systematic literature review, a large sample size. We validated the models using independent data from a different hospital, and we used a nonparametric statistical approach, including a machine learning component, to obtain unbiased estimates for variable importance. The high face validity of our findings, including the result of the practical application yielding similar frequencies, confirms that this modeling yielded conservative but robust predictors.

Our study had several limitations. Our models were built on data from only 2 tertiary-care university hospitals in Germany, albeit of high data quality; however, these findings may not apply to other hospitals. Furthermore, our analyses were based on data from EHRs that are collected for clinical and reimbursement purposes rather than for scientific use. Outcome assessment was conducted by a team of experienced physicians without interrater reliability testing. However, team members diligently communicated and resolved discrepancies during data collection to adhere to the predefined definitions. Our study only included cases of the current variant of concern and was conducted during a pandemic phase in which universal admission testing was performed in Germany, which resulted in a notable increase in the hospitalization rate. As SARS-CoV-2 and testing strategies evolve, other characteristics may become more important indicators. Particularly, viral load should be monitored during the entire stay to describe clinical disease progression. However, viral load data were not routinely collected and stored in the data warehouse of the hospital in a structured manner and could therefore only be included in the extended model. The observed signs and symptoms could also be attributed to concurrent infections with other respiratory agents, such as rhinovirus, which were not routinely tested. Nevertheless, all included patients presented with a positive PCR test for SARS-CoV-2, making it the most probable causative agent. Also, hospital-acquired COVID-19 as well as patients with a delayed onset were not considered. These patients might present with a different clinical picture warranting further investigation. Vaccination status is widely recognized as a significant factor influencing the risk of hospitalization and disease severity.^29^ Due to the substantial quantity of missing data, which may carry valuable information, we opted not to include this variable in our analysis. Finally, the presence of false-negative results cannot be ruled out and may have affected the completeness of our patient cohort. However, we anticipate the occurrence of false negatives to be minimal.

The presented algorithm yields an innovative parameter for assisting stakeholders in their political decisions. As a basis for calculating the hospitalization rate, it acts as an adaptive tool reflecting the existing needs in the current pandemic phase. Notably, incidental cases, while contributing to the hospital’s burden in terms of isolation and the need for protective measures for staff, can be more accurately managed when primary and incidental cases are differentiated. Moreover, it can be used in a modified version for current and future pandemic, epidemic and endemic situations all over the world.

## Supporting information

Strobl et al. supplementary material Strobl et al. supplementary material

## Supplementary material

For supplementary material accompanying this paper visit https://doi.org/10.1017/ice.2024.3
