# Supplementary material for: Differentiating patients admitted primarily due to coronavirus disease 2019 (COVID-19) from those admitted with incidentally detected severe acute respiratory syndrome corona-virus type 2 (SARS-CoV-2) at hospital admission: A cohort analysis of German hospital records
Source: Infect Control Hosp Epidemiol. 2024 Feb 14;45(6):746–53. doi: 10.1017/ice.2024.3 (PMC11102825; doi:10.1017/ice.2024.3)
Supplement: Strobl et al. supplementary material [file S0899823X24000035sup001.pdf]

Supplementary Material

Differentiating COVID-19 patients from incidental SARS-CoV-2 infection at hospital admission: a cohort analysis of German hospital records

Table of Contents

Data extraction 2

Results from the preparatory studies 3

 Admission diagnosis 3

 Further Criteria 4

Explanatory Variables 5

 Identified ICD-10-GM diagnoses 7

Baseline characteristics as row wise percentage 8

Discrimination plots 9

 Point-of-care model 9

Calibration plots 11

 Point-of-care model 11

 Extended point-of-care model 12

Variable importance 13

Data extraction

For the University Medical Center Goettingen (UMG) data set, patient records stored in the clinical information systems were used to extract a predefined set of variables.

For the University Hospital Würzburg (UKW) data set, pseudonymized data was extracted from the clinical information system SAP ERP 6.0 (SAP, Walldorf, Germany) combined with data from the COVID-19 surveillance database of the hospital.

Data from University Hospital Munich (UHM) were retrospectively analyzed based on the clinical routine data integrated in the Medical Data Integration Center (MeDIC). The related data were selected based on the criteria defined by HR2.0, as described in the manuscript, and were stored in a study specific data base.

Results from the preparatory studies

The following table gives an overview of the results of the three preparatory studies. For full information about these studies, we refer to:

Misailovski M, Koller D, Blaschke S, Berens M, Koester M, Strobl R, CODEX+ Monitor Study Group, Scheithauer S, Grill E (2023): Hospitalization due to COVID-19 versus incidental SARS-CoV-2 positivity: evidence for differentiation criteria from expert interviews and literature. submitted for publication.

Admission diagnosis

The following table displays the admission diagnosis measured according to the International Statistical Classification of Diseases and Related Health Problems (ICD-10-GM) considered in the preparatory studies.

Table S1a. All ICD-10 codes considered in the preparatory studies. X marks the diagnosis chosen ad indicative for a primary case

Admission diagnosis as ICD-10 code	Indicative
Viral and other specified intestinal infections (A08)	X
Infectious gastroenteritis and colitis, unspecified (A09)	
Other sepsis (A41)	
Erysipelas (A46)	
Anogenital herpesviral [herpes simplex] infections (A60)	
Viral infection of unspecified site (B34)	X
Other infectious diseases (B99)	X
Scabies (B86)	
Neoplasms (C00-D48)	
Diseases of the blood and blood-forming organs and certain disorders involving the immune mechanism (D50-D89)	
Endocrine, nutritional and metabolic diseases (E00-E90)	
Mental and behavioural disorders (F00-F99)	
Diseases of the nervous system (G00-G99)	
Postviral fatigue syndrome (G93.3)	
Diseases of the eye and adnexa (H00-H59)	
Essential (primary) hypertension (I10)	X
Acute myocardial infarction (I21)	
Pulmonary embolism (I26)	X
Stroke (I64)	
Acute upper respiratory tract infection (J06)	X
Viral pneumonia, not elsewhere classified (J12)	X
Acute pneumonia (J18)	X
Adult respiratory distress syndrome [ARDS] (J80)	X
Respiratory failure, not elsewhere classified (J96)	X
Other respiratory disorders (J98)	X
Diseases of the digestive system (K00-K93)	
Diseases of the skin and subcutaneous tissue (L00-L99)	
Diseases of the musculoskeletal system and connective tissue (M00-M99)	
Diseases of the genitourinary system (N00-N99)	
Pregnancy, childbirth and the puerperium (O00-O99)	
Congenital malformations, deformations and chromosomal abnormalities (Q00-Q99)	
Heartbeat disorders (R00)	
Cough (R05)	X
Dyspnea (R06)	X
Other symptoms and signs involving the circulatory and respiratory systems (R09)	X
Disturbances of smell and taste (R43)	X
Fever of other and unknown origin (R50)	X
Malaise and fatigue (R53)	X
Septic shock (R57)	
Systemic inflammatory response syndrome [SIRS] (R65)	
Multiple organ failure (R68)	
Injury, poisoning and certain other consequences of external causes (S00-T98)	
Diseases of uncertain etiology, assigned and unassigned codes (U07)	X
Multisystem inflammatory syndrome associated with COVID-19 (U10)	X
Isolation as a prophylactic measure for COVID-19 (SARS-CoV-2) (Z29)	
Undesirable side effects of using COVID-19 vaccines (U12)	
Carrier of infectious disease (Z22)	X

Further Criteria

The following table shows the further identified as potential relevant in the preparatory studies.

Table S1b. Criteria identified as potential indicative for a primary case.

**General criteria**
Age
Gender
Admission Ward
Number of SARS-CoV-2 vaccinations
Type of SARS-CoV-2 vaccinations
Recovery status
Period from last SARS-CoV-2 infection
Admission to intensive care unit
Admission via emergency department
**Therapy/Laboratory parameters**
ECMO therapy
Invasive ventilation
Therapy with oxygen mask/nasal oxygen cannula
Intensive care
Vasopressor therapy
Therapy with corticosteroids
Oxygen saturation
Blood pressure (systolic and diastolic)
Viral load
Full blood count
Differential blood count
**Comorbidities**
Heart diseases
Lung diseases
Kidney diseases
Cerebral circulatory disorders
Diabetes mellitus
Obesity/overweight
Peripheral Arterial Disease
Gastrointestinal disease
Dementia
Hemiplegia/paralysis
Malignancy/tumor
AIDS/HIV

Explanatory Variables

Table S1 describes all variables and their respective transformations together with their availability in UMG, UKW, and UHM.

Table S2. Potential predictors included for modelling. "x" indicates if available at the respective hospital

Variable	Description and Transformation	UMG	UKW	UHM
Gender	Biological sex as Male/Female	x	x	x
Age	Age at admission and categorized as infants (< 1), Pre-School (1 - 5), School (6 - 12), Adolescent (13 - 17), "Adults (>= 18)	x	x	x
Admission diagnosis(es)	ICD-10-GM codes at the category level of all admission diagnosis(es) indicative for a primary case<br><br>Essential (primary) hypertension (I10), Acute upper respiratory infections of multiple and unspecified sites (J06), Viral pneumonia, not elsewhere classified (J12), Pneumonia, organism unspecified (J18), Adult respiratory distress syndrome (J80), Respiratory failure, not elsewhere classified (J96), Cough (R05), Other symptoms and signs involving the circulatory and respiratory systems (R09), Disturbances of smell and taste (R43), Fever of other and unknown origin (R50), Diseases of uncertain etiology, assigned and unassigned codes (U07), and Multisystem inflammatory syndrome associated with COVID-19 (U10).<br><br>Viral and other specified intestinal infections (A08), Viral infection of unspecified site (B34), Other infectious diseases (B99), Pulmonary embolism (I26), Other respiratory disorders (J98), Dyspnoea (R06), Malaise and fatigue (R53), and Carrier of infectious disease (Z22).	x	x	x
Ward of admission	To harmonize medical admission ward of inpatient stay among the two hospitals we categorized the wards as follows:<br><br>• Non-surgical ward<br>• Surgical ward<br>• Intensive care unit	x	x	x
Admission via emergency department	Coded as "Yes" vs. "No"	x	x	x
Admission to intensive care unit	Coded as "Yes" vs. "No"	x	x	x
Discovery Time	Days from admission until positive SARS-CoV-2 result	x	x	
Vaccination status	„Unknown", „Not vaccinated", „Vaccinated"	x	x	
Discharge diagnosis(es)	ICD-10 codes at the category level of all discharge diagnosis(es)	x	x	x
Mortality status	Patient died during hospitalization	x	x	
Length of stay	Discharge date (date of death, if applicable) minus Admission date	x	x	x
Viral load	The logarithm of the quantity of the SARS-CoV2 virus in the test sample	x		
Therapy	The following therapy options were extracted from the patient records as „Conducted" vs. "Not Conducted". If no explicit information was given in the patient record, the therapy option was coded as "Not Conducted".<br><br>• Treatment in an intensive care unit (ICU)<br>• Extracorporeal membrane oxygenation (ECMO)<br>• Invasive ventilation<br>• Non-invasive ventilation<br>• Oxygen therapy via nasal canula or facemask<br>• Vasopressor therapy<br>• Corticosteroid therapy	x		
Comorbidities	The following comorbidities were extracted from the patient records as "Present" and "Not Present". Only comorbidities present before hospitalization were considered. If no explicit information was given in the patient record, the comorbidity was coded as "Not Present".	x		

Variable	Description and Transformation	UMG	UKW	UHM
	● Heart disease<br>● Peripheral arterial occlusive disease<br>● Lung disease<br>● Kidney disease<br>● Gastrointestinal disease<br>● Dementia<br>● Palsy/paralysis<br>● Diabetes mellitus<br>● Cancer<br>● Obesity/overweight*<br>● HIV/AIDS			
Reason for admission	The following diagnoses or symptoms, respectively, were extracted from the patient records as “Present” and “Not Present”. Only events or symptoms present upon hospitalization admission (defined as the first 24 hours after initial presentation) were considered. If not explicit or indicative information was given in the patient record, the event was coded as “Not Present”.<br><br>The following diagnoses were included as reasons for admissions:<br><br>● Acute pneumonia<br>● Pulmonary embolism<br>● Septic shock<br>● Acute respiratory distress syndrome (ARDS)<br>● ST-elevation myocardial infarction (STEMI)<br>● Non-ST elevation myocardial infarction (NSTEMI)<br>● Stroke or transient ischemic attack (TIA)<br><br>The following symptom was included upon admission:<br><br>● Dyspnea	x		
Clinical parameters	The following clinical parameters were extracted from the patient records and the database of the hospital laboratories. To handle missing values the variables were coded as follows:<br><br>• “Measured” and “Not measured”<br>• „Pathological“ and „Normal“<br><br>Classification was based on age-adjusted thresholds. Further information can be found in the electronic appendix.<br><br>The following parameters along with their units were measured on admission or within 24 hours after admission:<br><br>● Oxygen saturation (%)<br>● Systolic blood pressure (mmHg)<br>● Diastolic blood pressure (mmHg)<br>● High sensitive Troponin I (ng/l)<br>● D-Dimer (mgl/l FEU)<br>● C-reactive protein (CRP) (mg/l)<br>● Procalcitonin (µg/l)<br>● N-terminal pro b-type natriuretic peptide (NT-proBNP) (ng/ml)<br>● Hemoglobin (g/dL)<br>● Hematocrit (%)<br>● Erythrocytes (10^6/µl)<br>● Mean corpuscular volume (MCV) (fl)<br>● Mean corpuscular hemoglobin (MCH) (pg)<br>● Mean corpuscular hemoglobin concentration (MCHC) (g/dL)<br>● Platelets (10^3/µl)<br>● Leukocytes (10^3/µl)<br>● Lymphocytes (%)<br>● Monocytes (%)<br>● Eosinophils (%)<br>● Basophils (%)<br>● Neutrophils (%)	x		

* Patients with a body mass index of 30 kg/m² or more or with ICD-10 diagnosis E66 defined in the discharge letter were considered as overweight/obese.

Identified ICD-10-GM diagnoses

The following table shows the frequency and percentage of primary cases among the admission diagnoses defined as relevant.

Table S3. ICD-10 Diagnoses considered as relevant for distinguishing between primary and incidental cases

ICD-10 Diagnosis	Primary Cases
Acute obstructive laryngitis [croup] and epiglottitis (J05)	6 (100%)
Acute respiratory distress syndrome (J80)	22 (100%)
Fever of other and unknown origin (R50)	20 (100%)
Other respiratory disorders (J98)	10 (100%)
Viral pneumonia, not elsewhere classified (J12)	53 (93.0%)
Acute upper respiratory infections of multiple and unspecified sites (J06)	71 (91.0%)
Viral infection of unspecified site (B34)	23 (88.5%)
Cough (R05)	5 (83.3%)
Infectious gastroenteritis and colitis, unspecified (A09)	5 (83.3%)
Other and unspecified infectious diseases (B99)	5 (83.3%)
Viral and other specified intestinal infections (A08)	9 (81.8%)
Abnormalities of breathing (R06)	21 (80.8%)
Pulmonary embolism (I26)	4 (80.0%)
Convulsions, not elsewhere classified (R56)	7 (77.8%)
Carrier of infectious disease (Z22)	25 (75.8%)
Acute bronchitis (J20)	6 (75.0%)
Other disorders of fluid, electrolyte and acid-base balance (E87)	5 (71.4%)
Heart failure (I50)	4 (66.7%)
Transient cerebral ischemic attacks and related syndromes (G45)	4 (66.7%)
Malaise and fatigue (R53)	17 (65.4%)

Baseline characteristics as row wise percentage

The following table describes the characteristics of the patients included in the models with row wise percentages.

Table S4. Baseline characteristics of the study population from UKW and UMG presented as row wise percentages. Primary cases are those admitted to the hospital because of acute COVID-19. Incidental cases are those patients whose admission to the hospital was unrelated to their SARS-CoV-2 infection.

	Levels	All (n = 1150)	Primary Case (n = 462)	Incidental Case (n = 688)
Gender	Female	528 (46%)	216 (41%)	312 (59%)
Age (years; y)		49.5 (SD=28.5)	46.0 (SD=31.7)	51.8 (SD=25.9)
Age brackets (y)	Infants (< 1)	70 (6%)	51 (73%)	19 (27%)
	Pre-School (1 - 5)	83 (7%)	53 (64%)	30 (36%)
	School (6 - 12)	42 (4%)	20 (48%)	22 (52%)
	Adolescent (13 - 17)	32 (3%)	14 (44%)	18 (56%)
	Adults (>= 18)	923 (80%)	324 (35%)	599 (65%)
Ward of admission	Intensive care unit	164 (14%)	114 (70%)	50 (30%)
	Non-surgical ward	639 (56%)	232 (36%)	407 (64%)
	Surgical Ward	347 (30%)	116 (33%)	231 (67%)
Need for intensive care during hospital stay	Yes	147 (13%)	89 (61%)	58 (39%)
Mortality status	In-hospital mortality	54 (5%)	36 (67%)	18 (33%)
Length of stay (days)		7.7 (SD=12)	7.6 (SD=10.1)	7.8 (SD=13.1)
Vaccination status	No Vaccination	323 (28%)	187 (58%)	136 (42%)
	Vaccinated against SARS-CoV-2	607 (53%)	231 (38%)	376 (62%)
	Unknown	220 (19%)	44 (20%)	176 (80%)
Timing of RT-PCR test	Before admission	12 (1%)	4 (33%)	8 (67%)
	At admission	1026 (89%)	408 (40%)	618 (60%)
	<1-3 days after admission	112 (10%)	50 (45%)	62 (55%)
**Variables used in the extended model (UMG data with n = 344)**				
Oxygen therapy	Yes	56 (16%)	51 (91%)	5 (9%)
Logarithm to base 10 of viral load		5.9 (SD=2.2)	6.5 (SD=1.9)	5.0 (SD=2.4)
C-reactive protein (mg/l)		44.2 (SD=75.6)	47.5 (SD=77.8)	39.2 (SD=72.3)

*Abbreviations: Reverse transcriptase-polymerase chain reaction (RT-PCR); Severe Acute Respiratory Syndrome Corona-Virus type 2 (*SARS-CoV-2)

Discrimination plots

The figures S1 and S2 display the discrimination of the point-of-care model as ROC curves.

Point-of-care model

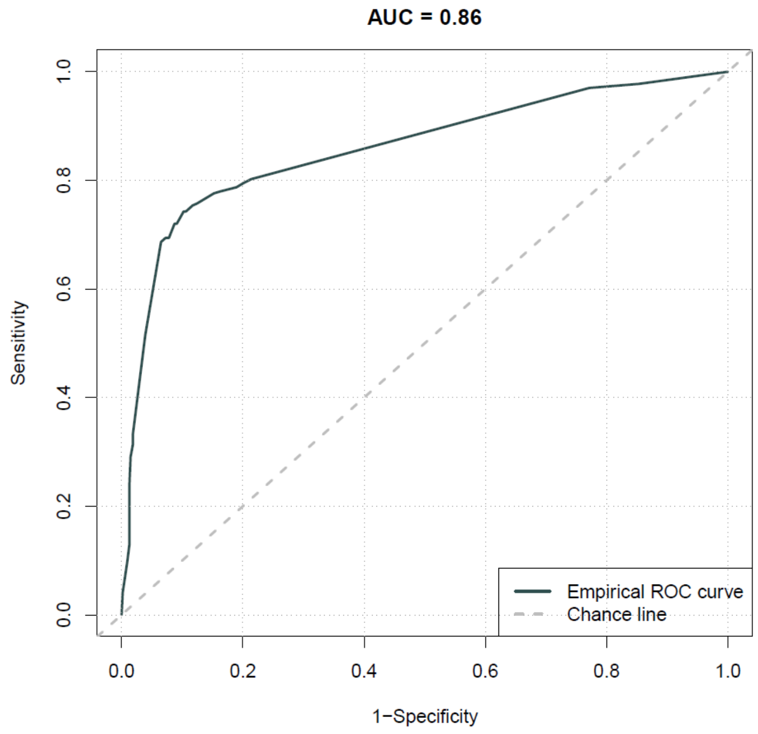

Figure S1 ROC curve for point-of-care run on the UKW learning data

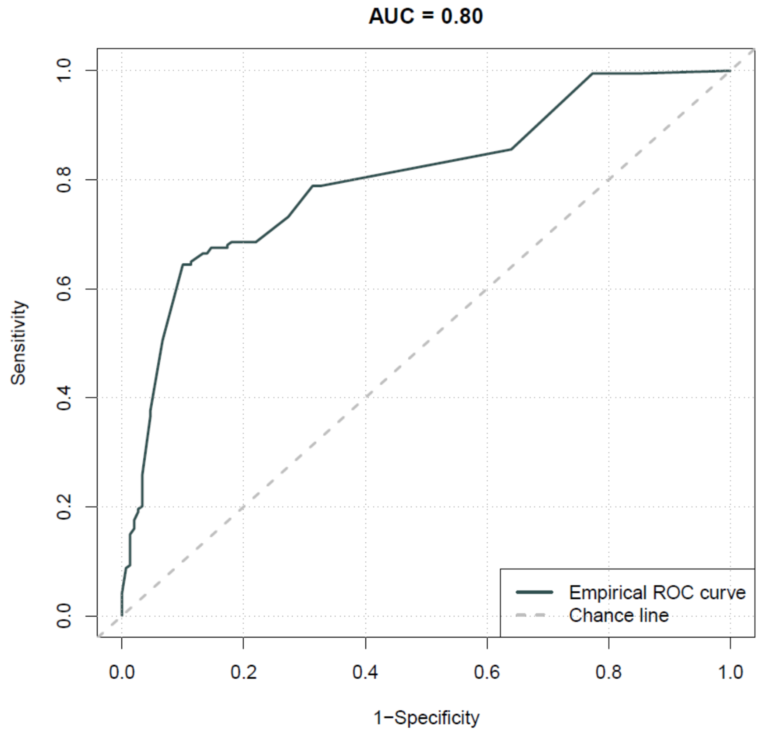

Figure S2 ROC curve for point-of-care run on the UMG validation data

Extended point-of-care model

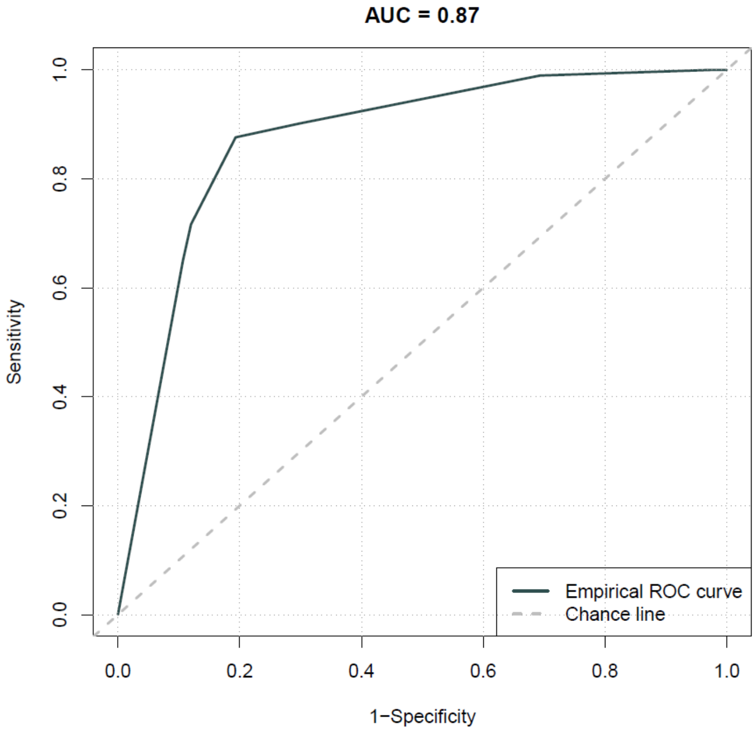

Figure S3 ROC curve for the extended point-of-care run on the UMG data set

Calibration plots

The figures S3 and S4 display the calibration of the point-of-care model by plotting the predicted probability against the observed relative frequency.

Point-of-care model

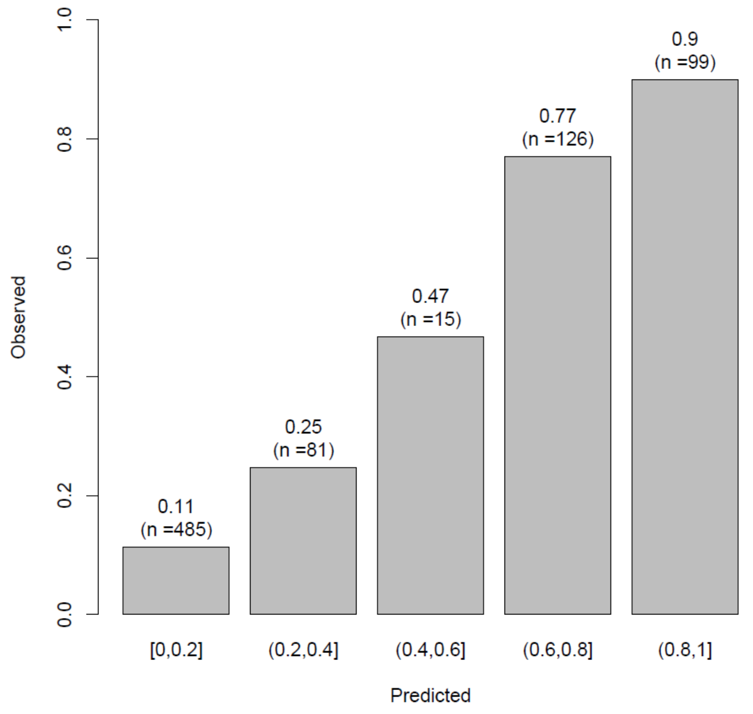

Figure S4 Non-parametric estimate of the association between the observed frequencies and the predicted probabilities of the point-of-care model run on the UKW learning data

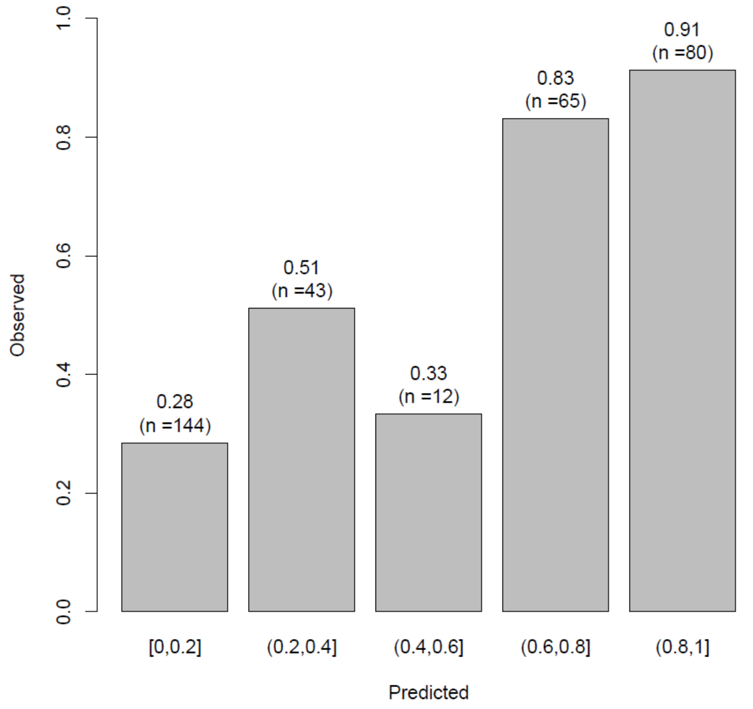

Figure S5 Non-parametric estimate of the association between the observed frequencies and the predicted probabilities of the point-of-care model run on the UMG validation data

Extended point-of-care model

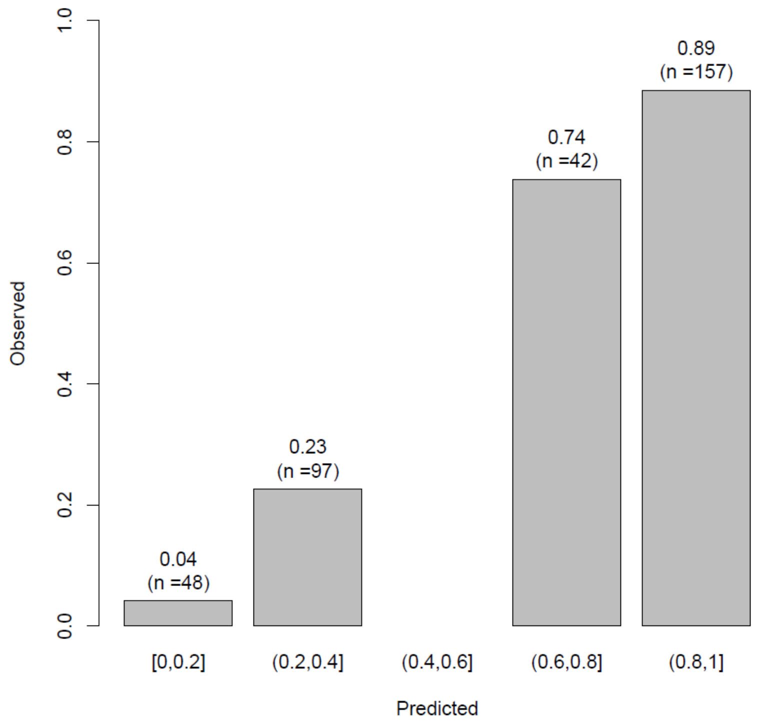

Figure S6 Non-parametric estimate of the association between the observed frequencies and the predicted probabilities of the extended point-of-care model run on the UMG data

Variable importance

The following table describes the variable importance of the 20 most important variables as identified by Random Forests.

Table S5. The 20 most important variables as identified by Random Forests

**Variable**
Admission Diagnosis (ICD-10)
Viral load
Leukocytes
C-reactive protein
Thrombocytes
Erythrocytes
Ward of admission
Hematocrit
Mean corpuscular haemoglobin (MCH)
Hemoglobin
Mean corpuscular hemoglobin concentration (MCHC)
Mean corpuscular volume (MCV)
Oxygen therapy
Acute pneumonia
Age
Sex
Kidney disease
Obesity
Cancer
Corticosteroid therapy
